# Milk Treatment of Disease

**Published:** 1884-08

**Authors:** James Tyson

**Affiliations:** Philadelphia, Pa.


### Selections.

---

### MILK TREATMENT OF DISEASE.*

BY JAMES TYSON, M. D., PHILADELPHIA, PA.

Journal of the American Medical Association.

In the course of a somewhat extended experience with what is known as the "milk cure," it has occurred to me that the growing popularity of this form of treatment demanded, at this time, some systematic consideration of the principles and practice on which its use is justified, as well as an outline of the class of cases in which it may be expected to be of advantage.

It is now a little more than eighteen years† since Dr. Philip Karell read his paper on the "Milk Cure" before the Medical Society of St. Petersburg.

To this paper I must refer for a large number of interesting historical facts, of both ancient and modern date, bearing on this subject, beginning with Hippocrates and coming down to the date of the paper itself. Among the diseases for which it had been recommended during this period, were phthisis, gouty affections, particularly articular, sciatica, leucorrhœa, hectic fever, dropsy, typhoid fever, intestinal obstruction, Bright's disease, intermittent fever, obesity.

Karell himself numbers the cases of successful treatment with milk "by hundreds." Dr. Inozemtseff, the author of a work on the "Milk Cure," published in Moscow in 1857, treated, with the help of his assistants, 1,000 cases. The former further says, "With regard to my own practice I have, after fruitlessly trying all sorts of remedies in many chronic and obstinate diseases, at last succeeded

---

*Read before the Section on Practice of Medicine and Materia Medica of the American Medical Association, May, 1884.

†On the Milk Cure by Philip Karell, M. D., Physician to His Majesty the Emperor of Russia. Translated from the author's manuscript by G. L. Carrick, M. D., Physician to the British Embassy at St. Petersburg. Edinburgh Medical Journal, Vol. xii, Part 1, p. 96, Aug., 1866.

in bringing the alimentary canal that seat of so many diseases, under my control. I did this by administering milk according to a new method." He says also, "After a great deal of experience, I have arrived at the conclusion, that in all dropsies, in asthma, when the result of emphysema and pulmonary catarrh; in obstinate neuralgia, when its cause lies in the intestinal canal; in diseases of the liver (simple hypertrophy and fatty degeneration), and generally in diseases when there is faulty nutrition, often a consequence of obscure sub-acute inflammation of the stomach or intestines, followed by affections of the nervous centers—in all these cases I consider milk as the best and surest of remedies. Even in those cases where the dropsy is the result of organic heart disease,or of old standing liver complaint, or of far advanced Bright's disease, I have seen very marked improvement take place, which also lasted a considerable time."

I have thus freely quoted Karell, because I believe the date of his paper marks an era in the history of the milk cure. He was instrumental in directing the attention of many to this method of treatment, and among them Prof. Niemeyer, who writes to him in 1861; "I thank you sincerely for having recommended the milk cure to me. I often resort to it and can praise it highly. If one were to acknowledge the existence of a number of diseases, the cause of which is not to be sought for in the remediable affections of certain organs, but rather in a perverse nutrition of which we are unable to define either the extent or nature, we must then admit the curative virtues of milk, and regard as a true advance in science, the discovery that this aliment is an innocent, and at the same time efficacious remedy, for producing a complete change of nutrition."

Referring to the extraordinary success claimed by some, Karell says he has no faith in so large a number of fortunate cases. He does not attempt to decide whether the beneficial influence of milk in certain illnesses is due merely to its nutritive qualities or some occult medicinal virtue. He simply calls attention to the fact that milk and chyle resemble each other very closely, insists that it must be taken at regular intervals under the direction of an experienced person, in doses from two to six ounces of *skimmed* milk, and that the best results are only obtained when the diet is an exclusive one. He further characterizes it as a regulator of nutrition.

My own experience with the milk treatment includes the following classes of cases: diabetes, calculous disease, Bright's disease, dyspepsia, obesity, and certain instances of the so called nervous prostration in women, in which its use formed a part of the treatment by rest, seclusion, massage, and electricity, and which has produced such satisfactory and widely known results in the hands of Dr. S. Weir Mitchell, of Philadelphia.

First, as to *diabetes mellitus*, it is now generally conceded that no measures are so efficient in removing the sugar from the urine, and relieving other symptoms, as the dietetic, and of the dietetic treatment none has been so promptly efficient in my hands as an exclusive milk diet. As to the degree in which relief is afforded, it must be admitted that a cure cannot always be guaranteed. The possibility of this depends upon the lesion at the bottom of the disease. But I add my testimony to that of Donkin and others to the effect that a certain number of cases are completely relieved, and that the symptoms do not return, after a cautious addition to the milk diet, and gradual substitution by nitrogenous, and, still later, even by starchy and saccharine foods. Other cases, again, show no sugar in the urine as long as the milk diet is continued, although this and other symptoms recur immediately that it is omitted and other food is substituted. In a third class of cases, sugar is much diminished, but does not disappear, and in such instances I have invariably found, in the most carefully conducted comparative experiments, that no other treatment is as efficient in diminishing the glycosuria. In a last and smallest number of cases, the milk diet is really badly borne, and it is impossible to carry it out; but in the large majority it is the most efficient remedy of which I know.

My method of administering milk in diabetes and indeed in all the conditions to which I will refer as adapted to it, is to begin with half a tumblerful, or four ounces of skimmed milk every two hours, for the first day, from which it is evident only thirty-two ounces, or a quart, can be given from 7 A. M. to 9 P. M. This is, of course, insufficient for an adult person, and even the second day I increase to six ounces every two hours, and after a day or two more to eight ounces, in which way sixty-four ounces, or two quarts are reached in the period named. This amount is quite sufficient for many persons of both sexes with small frames and light weight, but is quite insufficient for others, for whom the quantity may be increased by taking more at one time, or beginning a little earlier in the day and continuing later; or a glass or two may be taken during the night. Or, the interval may be increased to three hours, when still larger quantities must be taken at a time, until an amount sufficient to appease hunger or to maintain a good weight is reached. I

have known twelve and a half pints to be taken per day by a large man whose average weight was 195 pounds, but it is very seldom that so much is needed, and its ingestion becomes very inconvenient. When, however, such quantities are necessary, a part may be taken in the shape of curd, as suggested by Donkin. Thus, the man just referred to took the curd from four and a half to six pints, and drank the remainder of twelve and a half pints per day, he having found this much necessary in order to retain his weight while exercising actively during the day. While using eight to ten pints a day, part in milk and part in curd, he lost a half to three-fourths of a pound daily, although his health and strength continued perfect.

It is impossible to lay down a rule as to the quantity required in the twenty-four hours, but it may be roughly put at from five to ten pints for persons of medium stature, the larger quantity being necessary for those who are exercising, and the smaller for persons at rest.

The milk should be taken slowly, and not gulped in large amounts at a time. Not less than five minutes should be occupied in the drinking of a single glass of eight ounces. Nor should it be taken very cold. In summer the temperature should be about 60°, or a little below that of the surrounding atmosphere, and in winter it may be raised to the same temperature, or even slightly warmed, but it should not be boiled, unless diarrhœa is present.

Such a diet is, of course, quite compatible with health and strength for an indefinite time. And while I hardly advise that no attention be paid to the complaints of those who say that milk does not agree with them, I always insist on being allowed to settle that question myself by actual trial; and it is well known that where unskimmed milk is not well borne, producing, as it sometimes does, discomfort from flatulence or other cause, the skim-milk may be taken without causing any such sensations. Should the latter still disagree, which is very rarely the case, the liberal addition of lime water in the beginning, say but an amount equal to one-fourth, or even one-half the milk used, will often correct the difficulty; and later the milk can be gradually resumed in full strength. Occasionally, too, persons will complain of feeling weak upon a milk diet. This complaint, which is also often unfounded, may be met by increasing the amount prescribed.

A complaint of greater importance is the constipating tendency of a milk regimen, particularly at first. But this may be corrected by the daily use, if necessary, of one of the saline aperients, of which sulphate of magnesium is one of the best, or one of the aperient mineral waters, as Hunyadi Janos, or Friedrichshalle, or some one of the Saratoga aperient waters; or if these are insufficient, a pill of compound extract of colocynth, podophyllin and extract of hyoscyamus may be used; or a cup of black coffee in the morning may be sufficient. In many cases this symptom disappears after a time, and the whitish, almost odorless evacuations continue daily.

In diabetes mellitus, more than in any other conditions to which the milk treatment is applicable, it should be exclusive, at least at first. My plan is to continue the milk until sugar has been absent for from four to six weeks, after which I gradually add other foods, beginning with unskimmed milk, meats, oysters, fish, tomatoes, the green vegetables, gluten bread, fruits. The urine is tested after each addition of food, and if glycosuria is found, the article of food responsible for it is omitted for a time longer.

If I am asked the rationale of the action of skim milk in the treatment of diabetes, I am compelled to admit that I have no reason to believe it is directly curative. The most that can be said of it is that by furnishing a food which is non-irritating and easily assimilable, even as to its saccharine constituent, lactose, we give a rest to the starch and sugar assimilating apparatus, and allow the reparative tendency of nature to assert itself, and the hepatic or intestinal condition which causes the glycosuria, to disappear.

This is no time to discuss the pathology of diabetes, but there can be no doubt but that a certain number of cases consist in a simple organic or functional derangement of organs which only require to be let alone in order to restore themselves to a normal state, while the irritation caused by foods which can only be assimilated through their offices, must result in more or less permanent lesions. There are few who have not realized in some degree the suffering incident to the use of an inflamed muscle which prompts us to place it in a state of rest. The liver can make no appeal of this kind against its abuse; and whether the condition as the result of which it can not make the normal disposition of the glucose be primary to it, or secondary to a primary lesion elsewhere, the continued use of such foods can only aggravate both primary and secondary lesions.

Now, the skim-milk furnishes a food of a kind which gives the liver a thorough rest—and a more complete rest than any other diet as yet suggested. This, I am forced to admit, is the only way in which I conceive it acts in the cure of diabetes. Hence it is that there are certain cases due to lesions remote from the liver, which are themselves incurable, either spontaneously or through direct treatment, which milk cannot reach; such, I believe, are some forms of pancreatic disease. In these cases, the lives of the patients are prolonged by any diet which furnishes the requisite reparative and force-producing elements in an available shape. In such case, a starchy and saccharine food is wasted, simply because the resulting grape sugar passes directly through the system. Milk and the albuminous foods, not being converted to any extent into grape sugar, are still available as force producers, and thus defer the unfavorable termination, or, in a word, keep the diabetic from starving.

It may also reasonably be asked, why is skim-milk superior to unskimmed? I believe any superiority consists in the fact that milk deprived of its cream is simply more easily assimilable than unskimmed milk, and therefore less likely to disturb digestion. There are probably none here who have not heard the complaint from certain patients that a milk diet makes them bilious, by which is merely meant that it causes indigestion. And there are doubtless many who have found the substitution of skimmed milk to remove these symptoms. I have, too, quite frequently observed that whatever food produces indigestion in a diabetic is apt to cause an increase in the glycosuria, independent of its composition. and I have sometimes thought that the use of the curd I have alluded to causes a slight return of glycosuria from this cause.

On the other hand. as already stated, I am quite in the habit of gradually adding unskimmed milk to the skimmed in cases of diabetes, and where there occur no symptoms of indigestion, I have continued it before adding other articles of food. In like manner, peptonized skimmed milk in which the casein is partly digested, is even more easily assimilated than the non-peptonized.

A second class of cases, in which the skim-milk has been of signal usefulness in my experience, and in which its results are capable of a more rational explanation than in diabetes. are cases of *uric acid gravel.* I have yet to see an instance of persistent uric acid sediment, in which the exclusive use of milk was not followed, sooner or later, by a total disappearance of the deposit, while I have known its persistent use to be followed by the easy discharge of uric acid calculi of considerable size, *per urethram.*

The rationale of these results has been made perfectly plain by the chemical study of the urine of persons on such a diet, by Dr.

John Marshall, of the University of Pennsylvania, undertaken at the suggestion of Dr. Mitchell, a year ago, and repeated recently for me. It is rather singular that, although skim milk has been used for so many years in diseases involving a study of the urine, examinations of this kind were not previously made. The most important result of these analyses, is the fact that in the total twenty-four hours' urine of persons on the skim milk, *no appreciable amount of uric acid could be found.*

Here, too, as might have been expected. the same results obtained, whether the patient used a diet of skimmed or unskimmed milk. Dr. Marshall examined for me for three days in succession, the urine of Mr. M., who had been for several weeks upon a diet of unskimmed milk. The quantity of milk ingested during these three days, was on the first day six pints, or 2,838 c.c.; on the second day the same, and on the third day five pints, or 2,365 c.c. The quantity of urine passed was, on the first day 1,356 c c.; on the second day 1,580 c.c., and on the third day 1,070 c.c. The total urea in the first was 31.59 grams, in the second 27.96, and in the third 19.68. *Uric acid was not present in recognizable quantities— i. e.* 500 c.c. of each sample, evaporated to dryness and properly extracted, gave no reaction with the murexid test. Dr. Marshall says further: "Uric acid probably might be found if five or six litres of the urine in question were evaporated to dryness and tested; most likely, however, only a trace."

Here, then, we have a simple rational treatment for uric acid gravel, and as successful as it is simple. There seems, too, to be no reason why the milk should be skimmed for this use, rather than unskimmed, while the latter has the advantage that a smaller amount is needed to satisfy hunger and keep up the weight of the body.

As to the propriety of an exclusive milk diet in these cases of uric acid gravel, it may be determined by experience. While it may not be necessary in every case, in some it is absolutely so. In the case of Mr. M., who had several severe attacks of nephritic colic, and who, under ordinary mixed diet, as constantly passes a large daily amount of uric acid, as he is constantly in discomfort, the addition of bread only to a meal, causes a return of uric acid, while a pure milk diet is unattended by such discharge.

While an exclusive milk diet is so efficient in cases of pure uric acid gravel, it is not to be especially recommended in *phosphatic calculus.* Indeed, it is rather contra-indicated. For the effect of a milk diet is to alkalize the urine, and therefore to tend to maintain a phosphatic sediment.

While nothing can be hoped for in respect to solvent action, or tendency to solvent action upon *oxalate of lime* sediments, by urine derived from a milk diet, yet, as it is commonly supposed that the same conditions of system which tend to produce uric acid are those which produce oxalate of lime, it may be expected that milk treatment will also be useful in the oxalate of lime tendency.

The third set of diseases in which I have found the milk treatment especially useful, are certain forms of *Bright's disease.* In these, more discrimination has to be exercised than in any of the foregoing. In general, it may be said that it is in the contracted kidney of interstitial nephritis that milk is most useful, the headache, nausea, vertigo, and "fullness" in the head, and the palpitations which are so often very annoying symptoms, are frequently relieved by it. Here, too, the skimmed milk is especially indicated, because anything which increases the labor of digestion aggravates the symptoms above mentioned as characteristic. It would seem, too, that those suffering with contracted kidney are better when the blood is not too good in quality or too rich in solids. On the other hand, it is not so necessary that the diet should be exclusively milk, and a sufficient quantity of bread and vegetables may be permitted to break up the monotony, while meat, eggs, and all foods rich in albumen should, as a rule, be prohibited. In this disease, especially, the constipation incident to a milk diet should be guarded against,

In parenchymatous nephritis and amyloid kidney I have found milk less useful, while it is still a very convenient and suitable diet, often producing diuresis and relieving dropsies. When a diuretic effect is desired, buttermilk is to be preferred, while it is as easily assimilated as skim-milk, and often more palatable.

That a milk diet should be useful in certain cases of *gastro-intestinal disease*, is generally recognized. There are two classes of cases for which it is adapted. The first is that represented by the ordinary dyspeptic, whose symptoms are sometimes dispelled as by magic by a pure skim-milk diet systematically carried out, and modified by the free addition of lime water. In a few instances of ordinary dyspepsia I have found the use of milk to produce flatulence, and to be altogether illy borne, but this tendency is corrected by peptonizing the milk.

At the same time, I cannot too strongly recommend in all cases of simplé dyspepsia, where other measures have failed, a trial of the milk treatment in the systematic manner described, and modified as the peculiarities of the special case may demand, and especially in the event of failure with simple skim-milk, that the peptonized milk be tried.

But it is more particularly in the treatment of organic diseases of the gastro-intestinal tract that the bland and unirritating qualities of milk diet are indicated. In gastric ulcer the use of no other food than peptonized milk should be permitted. In chronic enteric disease of both small and large intestines, and particularly of the latter, it is very doubtful whether it is in our power, by therapeutic means, to directly affect the diseased parts. Our treatment must therefore consist in such measures as will favor nature's inherent tendencies to restore lost tissue. This can only be done by placing the patient on a diet which is non-irritating, leaves little waste, and at the same time makes the smallest demand upon the digestive function. To accomplish this, it is necessary to obtain his consent to the use of a diet of peptonized milk for an indefinite period. For no promises can be made as to the date at which other food dare be permitted. The uninterrupted presence of natural stools, and the continued absence of pain, are the only reliable criteria. To be able to carry out such a treatment satisfactorily, it is absolutely essential to secure the confidence of the patient. The process of obtaining this depends largely upon the personal qualities of the physician, and the ability to be able to speak authoritatively, but when the probability of almost certain recovery is held out, and it is shown that the good effects of weeks of treatment are undone by so simple a solid as a small piece of bread, the battle is half won. Particularly in those cases of irritation of the large bowel, attended by the discharge of mucus or mucus-casts, may we expect from the milk treatment the most satisfactory results, while success by local treatment is very rarely attained. The use of milk is rational, and consistent with what we know of the essential conditions of growth and repair.

I will allude to but one more use of the milk treatment, referring to Dr. Mitchell's little book on "Fat and Blood" for a better exposition than I can give of its use in the class of cases in the treatment of which he has earned so much reputation. The purpose to which I will refer is that for the removal of excessive *obesity*, and for this a skimmed milk is necessary. No treatment which has ever been suggested, is half so efficient. I have seen a lady disposed to obesi ty, who, in consequence of a more than usually quiet summer, and the liberal use of food, return to the city so much increased in flesh that she could not put on the clothing which a few months before was worn with ease, and suffering with a shortness of breath which made walking any distance almost impossible, resume, in the course of two weeks' use of skim-milk, her usual weight and state of comfort. In these cases a pint of milk may be taken every four hours, or fifty-six ounces in the day, with directions to take more if hunger is not appeased. It is scarcely likely that even with this freedom too much can be taken to prevent the desired reduction in weight. It will be remembered that I referred to the case of a large man who was able to retain his weight upon twelve and a half pints a day, but when consuming ten pints daily, lost half a pound each day.

It may not be proper in all instances to reduce the weight so rapidly as can be done upon a skim-milk diet, especially when the subject is active or hard working, but it is to be remembered that obese persons are not usually of this kind, and that activity is facilitated by some loss of flesh. It is to be remembered, too, that the fat they carry is simply stored up in the subcutaneous tissue, because it is more than enough to supply the force-demands of the human machine, and has really had no use until the requisite daily amount of oxidizable material is not supplied in the daily food. Hence it is that when the weight is reduced approximately to a certain standard ratio to height, unskimmed milk or other articles of food should be judiciously added to furnish sufficient oxidizable material. But it is an interesting and important fact that when a certain stage of reduction is attained, further falling off, even with a moderate amount of milk, ceases, or takes place so slowly that it is easily made up by additions to the diet, to which of course there is no objection at this stage in the condition under discussion.

